# Membrane Lipids in Epithelial Polarity: Sorting out the PIPs

**DOI:** 10.3389/fcell.2022.893960

**Published:** 2022-05-31

**Authors:** Katlynn Bugda Gwilt, Jay R. Thiagarajah

**Affiliations:** Division of Gastroenterology, Hepatology and Nutrition, Boston Children’s Hospital, Harvard Medical School, Boston, MA, United States

**Keywords:** polarity, phosphoinositide, epithelial, apical, PI-kinase, phosphatase, TTC7, glycosphingolipid

## Abstract

The development of cell polarity in epithelia, is critical for tissue morphogenesis and vectorial transport between the environment and the underlying tissue. Epithelial polarity is defined by the development of distinct plasma membrane domains: the apical membrane interfacing with the exterior lumen compartment, and the basolateral membrane directly contacting the underlying tissue. The *de novo* generation of polarity is a tightly regulated process, both spatially and temporally, involving changes in the distribution of plasma membrane lipids, localization of apical and basolateral membrane proteins, and vesicular trafficking. Historically, the process of epithelial polarity has been primarily described in relation to the localization and function of protein ‘polarity complexes.’ However, a critical and foundational role is emerging for plasma membrane lipids, and in particular phosphoinositide species. Here, we broadly review the evidence for a primary role for membrane lipids in the generation of epithelial polarity and highlight key areas requiring further research. We discuss the complex interchange that exists between lipid species and briefly examine how major membrane lipid constituents are generated and intersect with vesicular trafficking to be preferentially localized to different membrane domains with a focus on some of the key protein-enzyme complexes involved in these processes.

## Introduction

Epithelial cells that line mucosal surfaces mediate many of the interactions between biological organisms and the outside environment. In the intestine, this includes a remarkable multi-functional remit, involving nutrient gathering via selective permeability, environmental sensing and surveillance, and defensive barrier function. A critical architectural feature that underlies these functions is the formation of cellular asymmetry or polarity, which consequently allows for vectorial movement of proteins, nutrients, ions and fluid across the epithelium. Epithelial cells contain two distinct plasma membrane domains, organized to direct vectorial transport and partition selective functions within the cell. Basolateral (or organism facing) membrane domains interface with the underlying tissue including the circulatory, lymphatic, immune and nervous systems in higher organisms, with the lateral aspect of this membrane mediating interactions between neighboring epithelial cells. Apical plasma membrane domains face the exterior lumen, and are primarily responsible for fluid, electrolyte and nutrient absorption or secretion, as well sensory and defense functions.

The development and maintenance of epithelial cell polarity has been the subject of extensive investigations in a variety of model systems (Mathan et al., 1976; Madara et al., 1981; Ng et al., 2005; Gassama-Diagne et al., 2006; Martin-Belmonte et al., 2007; Bryant et al., 2010; Alvers et al., 2014; Bryant et al., 2014; Román-Fernández et al., 2018). Studies have largely focused on the mechanisms and sequence of events by which cellular proteins are involved in the development of polarity. At a cellular level, epithelial polarity begins with initial cell division and cytokinesis, generation of an unpolarized cell, followed by the arrangement and rearrangement of specific membrane domains that eventually become defined apical and basolateral membranes (Martin-Belmonte et al., 2007; Román-Fernández et al., 2018). Several key protein complexes including the Par, Crumbs and Scribble complexes have been shown to be important in formation and maintenance of the intercellular junctions that define the boundary between apical and basolateral membranes. Although numerous studies have focused and coalesced around a set of core polarity proteins, there remains significant variations in both the key proteins involved in different epithelia such as the kidney vs. the intestine (Zegers et al., 2003; Roignot et al., 2013; Rodriguez-Boulan and Macara, 2014; Jewett and Prekeris, 2018) as well as between organisms (Mathan et al., 1976; Madara et al., 1981; Ng et al., 2005; Yan et al., 2011; Alvers et al., 2014).

Lipids are the major constituents of cellular membranes and studies, primarily in cyst models systems, have highlighted their importance as primary drivers in organizing and defining the apical and basolateral membranes in mammalian epithelia. In cells, several different classes of lipids exist that help both define and functionally separate membranes within organelles, endosomal compartments and the plasma membrane as well as between specific nanodomains within membranes (van Meer et al., 2008). These include phosphoinositides (PIs), phosphatidylserine, glycosphingolipids and cholesterol. An increasing body of literature suggests that these lipids serve specific and critical functions during polarization of the epithelium, including directing vesicular trafficking and the subsequent destination of protein constituents within the distinct compartments of the plasma membrane (Shewan et al., 2011; Roignot et al., 2013; Hammond and Hong, 2018). In this review, we broadly describe the evidence for a primary role for membrane lipids in the generation of epithelial polarity, concentrating principally but not exclusively on intestinal epithelia. We present a brief overview of tissue morphogenesis and the protein complexes primarily involved in polarity generation, and refer the reader to excellent and comprehensive reviews of these topics elsewhere (Zegers et al., 2003; Bryant and Mostov, 2008; Roignot et al., 2013; Bryant et al., 2014; Rodriguez-Boulan and Macara, 2014). We review the complex interplay and interchange that exists between lipid species and describe how major membrane lipid constituents are generated, intersect with vesicular trafficking, and are localized to different membrane domains; focusing on key protein complexes involved these processes. Finally, we review the existing evidence for specific membrane lipid species as drivers of apico-basolateral polarity and highlight key areas requiring further research.

### Overview of Intestinal Epithelial Morphogenesis

The formation of epithelial polarity is a critical process that underlies tissue morphogenesis in hollow lumen organs such as the intestine. The creation of tubes during development and tissue maintenance occurs at multiple levels within the intestine, in relation to the formation of a central intestinal lumen as well as various glandular structures. Broadly, there are a number of different models of lumen formation that exist across tissues, species and organ systems (Schlüter and Margolis, 2009; Sigurbjörnsdóttir et al., 2014; Jewett and Prekeris, 2018). Epithelial budding, a process that occurs during the formation of the mammalian lung and kidney and in *drosophila* salivary glands, trachea, and hindgut (Jewett and Prekeris, 2018), involves the outgrowth of a small subset of cells initially growing in a continuous layer to form a budded structure with a central tubular blind-ended lumen. Another process termed cavitation results from the apoptosis and subsequent loss of inner cells within a larger mass of cells, allowing for the clearing of a central luminal space and is exemplified by mammary duct formation (Jewett and Prekeris, 2018). The *de novo* generation of epithelial tubular structures has largely been ascribed to a process termed ‘cord hollowing” that has been extensively studied *in vitro* using 3-dimensional cyst models. The basis of this model involves the coordination of multiple cellular processes including polarization of epithelial cells, *de novo* apical membrane biogenesis, intracellular trafficking to a prospective apical domain, lumen enlargement and, in some cases, epithelial remodeling (Jewett and Prekeris, 2018).

Tubulogenesis and lumen formation in the intestinal epithelium is thought to broadly occur through a modified cord hollowing process requiring membrane polarization followed by initial generation of a multi-lumen structure that subsequently coalesces into a single lumen and tubular structure. There are several lines of evidence for this model, although many of the molecular details and particularly the role of membrane lipids remains to be fully elucidated. Important early studies in the rat intestine showed that the formation of the fetal duodenum occurs in several steps, with polarization of cells within a multicellular layer of stratified epithelium, followed by formation of multiple lumens between cells within this layer, and finally reorganization of these luminal structures into a single continuous lumen (Mathan et al., 1976; Madara et al., 1981). This same phenomenon has also been carefully described in the developing zebrafish intestine, with the initial polarization of epithelial cells leading to an intermediate multi-lumen structure and subsequent fusion of adjacent lumens (Alvers et al., 2014).

At the tissue level an integration mechanism termed radial intercalation (Sedzinski et al., 2016) has been proposed for re-organization of cells and lumens during tubular morphogenesis at barrier surfaces, at least in *Xenopus* and *Drosophila*. Radial intercalation involves the movement of basal cells toward an existing primary lumen, via rearrangement and expansion of adjacent cells. Recent high-resolution studies suggest that this process may describe aspects of tube formation in the intestine (Moreno-Roman et al., 2021). At the cellular level, the reorganization and fusion of secondary lumens has been proposed to critically rely on intracellular endosomal trafficking events driving sorting of membranes and consequently, membrane proteins, from the basolateral surface to the remodeled apical surface. These trafficking endosomes are typified by the presence of Rab11a GTPase, a well-described cell membrane protein that marks endosomes responsible for polarized cellular trafficking. Loss of Rab11a or its associated effector protein FIP5 function, and subsequent loss of endosomal sorting to the developing apical membrane results in the formation of multiple lumen structures in cyst cultures (Willenborg et al., 2011). Other studies have also highlighted the importance of junctional proteins (Odenwald and Turner, 2017; Buckley and Turner, 2018) as well as the correct orientation of critical ion and fluid transport proteins for correct lumen formation *in vitro* and *in vivo* (Bagnat et al., 2007; Alvers et al., 2014; Blasky et al., 2015).

As postulated by the cord-hollowing model, cell division events are linked to the first signs of apical and basolateral polarity establishment. The initial organization of the apical membrane occurs in the two-cell state following cell division and the delivery of key proteins to the apical membrane via vesicular transcytosis. This allows for the establishment of a transitional environment at the nascent apical membrane that has been termed the apical membrane initiation site (AMIS). The AMIS then progresses into a stage termed the pre-apical patch (PAP), where junctional membrane proteins begin to demarcate the separation of the apical and basolateral membrane (Zegers et al., 2003; Bryant and Mostov, 2008; Román-Fernández et al., 2018). Lumen initiation occurs at the PAP and paracellular ion transport and subsequent fluid accumulation enlarges the immature lumen allowing for expansion (Martin-Belmonte et al., 2007; Román-Fernández et al., 2018). Final coalescence of the multiple lumen intermediate state formed in the *in vivo* intestine appears to occur by rearrangement of basolateral proteins together with expansion of the apical membrane, with the Hedgehog signaling pathway implicated (Alvers et al., 2014) as well as apical-basolateral transcytosis. While the initial studies in the zebrafish intestine identified some of the key players in this process, the role of membrane PIs and PI-generating enzymes remain to be fully explored.

### Organization of Protein-Complexes During the Generation of Epithelial Polarity

**FIGURE 1:**
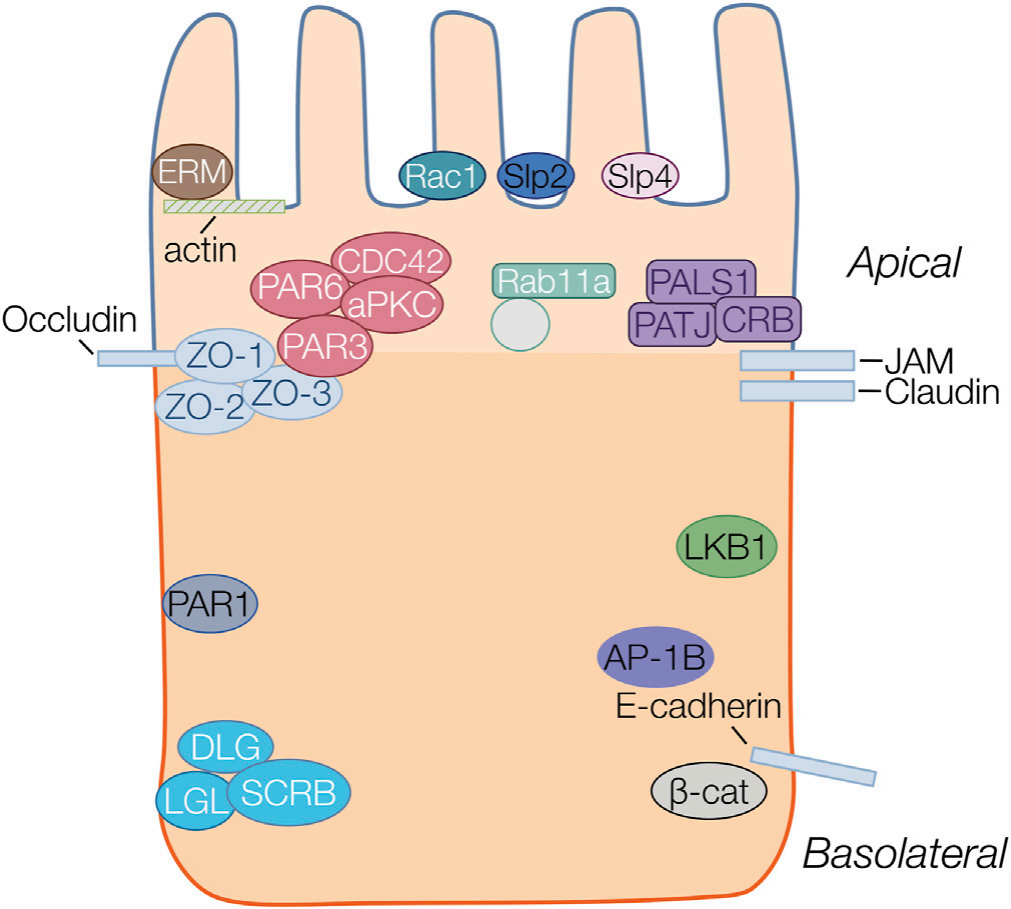
Protein complexes important in epithelial polarity. Proteins belonging to the same family or complex are represented in the same color. Four major polarity complexes are highlighted: the apically localized ERM (brown), Crumbs (Crb/Pals1/Patj) (purple) and Par complex (Par3/Par6/Cdc42/aPKC) (red) and the basolateral Scribble (Scrb/Lgl/Dlg) (aqua). The basolateral proteins Par1 (blue), LKB1(green) and AP-1B (purple) are also highlighted. Structural proteins including actin, and junctional proteins occluding, JAM, claudin and E-cadherin are all represented as rectangles. Par3 localizes to the cytoplasmic scaffolding Zonula Occludens (ZO) protein anchored to the membrane by occludin at the apical junctions. The ERM proteins provide anchorage to actin filaments at the apical membrane. Synaptotagmin-like proteins (Slp2 (navy) and Slp4 (pink) are anchors for vectorial transport towards the apical membrane. Transcytotic vesicles, transiting from basolateral to apical surfaces are marked by Rab11a to deliver and maintain the recycling of apical membrane proteins at the apical surface.

Despite differences in cellular structure and function, many aspects of the protein machinery involved during polarization in epithelia are highly conserved across species and have been the topic of several extensive reviews (Roignot et al., 2013; Rodriguez-Boulan and Macara, 2014; Hammond and Hong, 2018; Polgar and Fogelgren, 2018). Three major “polarity complexes” (Figure 1) have been characterized in studies and play key roles in orchestrating cell polarization. The major complexes include the Par complex located at tight junctions, the Scribble complex at basolateral membranes and the Crumbs complex at the apical membrane and junctions. The localization of these complexes during the development and establishment of apico-basolateral polarity is critically dependent on association and binding with underlying membrane lipids, and in particular specific PIs (discussed in detail in the next sections).

The initial formation of the apical membrane (AMIS) is characterized by movement and ultimately sequestration of polarity complexes to distinct membrane domains. The Par complex which includes various Par proteins (Par1,4,3,6), as well atypical protein kinase C (aPKC) and GTPase CDC42, is found at the early apical membrane before transitioning to its final location at tight junctions by interactions with ZO proteins, and forms the boundary between apical and basolateral membranes in terminally polarized cells (Macara, 2004; Krahn et al., 2010; Rodriguez-Boulan and Macara, 2014; Hammond and Hong, 2018; Jewett and Prekeris, 2018). The Par complex plays a central role in polarity development, primarily through recruitment of aPKC to junctions and subsequent phosphorylation of other complex members (Crumbs and Lgl), as well as interaction with CDC42. A critical aspect of Par protein complex localization occurs via various PI binding domains, which likely facilitates changes in localization through the course of emerging polarity. The Crumbs complex has also been shown to be an important component in the formation of the apical membrane. The Crumbs complex is found apically sequestered and is concentrated at tight junctions, where it interacts with the PALS1 adapter protein and PATJ, to interact with tight junction proteins. The Crumbs complex inhibits Rac1 mediated activation of PI3kinase (PI3K), allowing for the formation of the apical membrane domain (Chartier et al., 2011). Rho family GTPases, including CDC42, Rac1 and RhoA (Iden and Collard, 2008; Rodriguez-Boulan and Macara, 2014; Blasky et al., 2015), play a role in polarity development by control of cytoskeletal rearrangement. RhoA controls the actin-myosin filament assembly whereas Rac1 and Cdc42 organize microtubule and actin assembly at the cell membrane (Iden and Collard, 2008). Another group of important proteins involved in AMIS formation are the synaptotagmin-like proteins such as Slp2a, which are thought to cluster at the AMIS by interacting with PIs (Gálvez-Santisteban et al., 2012).

Following initial apical membrane specification, the formation of intercellular junctions including tight, adherens and gap junctions are crucial for partitioning of apical and basolateral membrane domains and maintenance of polarity. Tight junctions are belt-like structures that form a selectively permeable barrier separating apical from basolateral surfaces. Tight junctions are composed of a variety of structural and cytoskeletal connecting proteins such as the occludins, claudins, and junction adhesion molecules (JAMs), in addition to zonula occludens (ZO) proteins (Buckley and Turner, 2018). Following apical localization of Par3, cell-cell contacts are generated by enriching E-Cad, JAM and ZO1 at the membrane. These contacts activate Rac1 and RhoA to allow formation of cell-junctions (Ferrari et al., 2008). Tight junction maturation and maintenance is intimately correlated with the localization of the Par complex and by activation of aPKC (Lin et al., 2000; Schlüter and Margolis, 2012).

Vesicular trafficking is thought to play a major role in correctly localizing proteins to apical and basolateral membrane. The plasma membrane and intracellular organelle membranes are in constant flux with one another via endocytosis, transcytosis, vesicular recycling at apical and basolateral domains, and secretion from the Golgi to the plasma membrane (Blasky et al., 2015; Garcia-Castillo et al., 2017; Jewett and Prekeris, 2018). Multiple proteins are involved with the vesicular trafficking of apical and basolateral protein cargoes within the cell including small Rab-GTPases (i.e. Rab11a) as well as the clathrin adaptor proteins. The clathrin adaptor protein (AP) complexes have shown to be important in the sorting of cargoes within the cell (Bonifacino and Traub, 2003). Epithelial cells co-express the epithelial cell specific AP-1B complex in addition to the closely related ubiquitously expressed AP-1A complex. In epithelial cells, AP-1B mediates basolateral trafficking of polarity proteins from recycling endosomes, while AP-1A localizes at the trans-Golgi network and in early endosomes (Fölsch et al., 2003; Fölsch, 2005) AP-1A has been implicated in some instances to direct cargoes to both the apical and basolateral membranes (Gravotta et al., 2012; Gravotta et al., 2019).

Finally, the Ezrin, Radixin and Moesin (ERM) complex of proteins, which link membranes with the cytoskeleton, are important for localization of apical membrane proteins. Evidence for the importance of the ERM proteins in cellular polarity, and tissue morphogenesis come from studies where loss of function in any of the ERM proteins leads to aberrant polarity and lumen formation (Fehon et al., 2010). PIs have been shown to play an important regulatory role in the correct localization of the ERM complex (Michie et al., 2019). ERM also modulates the positioning and activation of apical membrane proteins responsible for fluid secretion, including NHERF (Na+-H+ Exchanger Regulatory Factor) (Terawaki et al., 1993; McClatchey, 2012), NHE3 (Zhao et al., 2004; Fehon et al., 2010), and NHERF/CFTR (McClatchey, 2012), as well as the proper translocation and insertion of H-K-ATPase vesicles in the membrane (Zhu et al., 2010).

As briefly outlined above, the protein complexes involved during polarity formation have been extensively studied with a broad set of main players now well established. The regulation of sorting and membrane localization of these protein complexes is thought be directed by the underlying membrane lipid environment, and in particular the presence and interconversion of PIs.

### Key Lipids Involved in the Development of Epithelial Polarity

**FIGURE 2:**
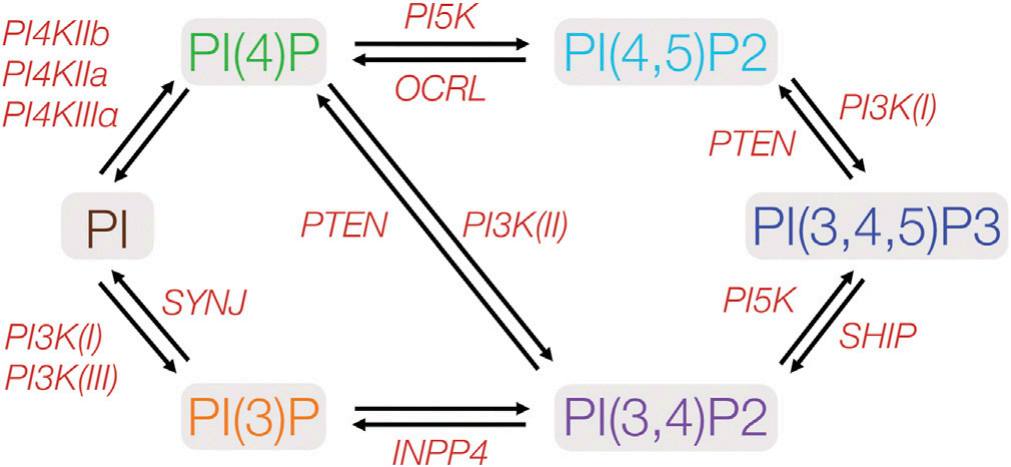
Metabolic pathways of phosphoinositides implicated in cell polarity. PIs may be phosphorylated at any of the three hydroxyl groups on the inositol ring. Protein kinases and phosphatases involved in PI phosphorylation and dephosphorylation are indicated in red. PIxK- phosphoinositide x-kinase; OCRL- Inositol polyphosphate-5-phosphatase; SYNJ-phosphoinositide 5-phosphatase; INPP4- inositol polyphosphate-4-phosphatase; SHIP- Src homology 2 domain containing inositol polyphosphate 5-phosphatase. PTEN- phosphatase and tensin homolog.

**FIGURE 3:**
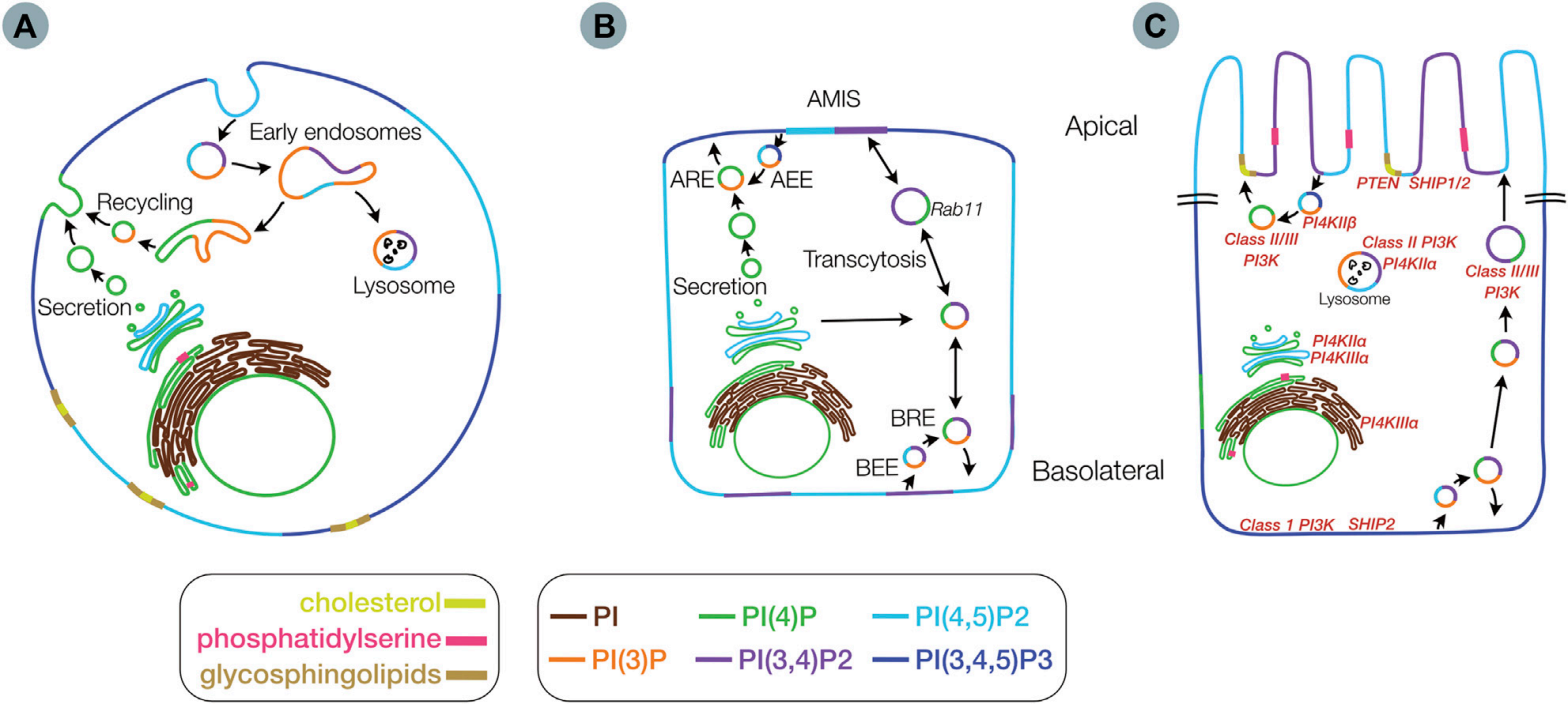
Membrane distribution of lipids involved in epithelial polarity. Membrane distribution of phosphoinositides (PIs), phosphatidylserine, glycosphingolipids and cholesterol in an unpolarized cell **(A)**, in a partially polarized cell **(B)**, with an apical membrane initiation site (AMIS), and in a fully polarized cell **(C)** including locations for key kinases and phosphatases (red) Inset legend indicates color scheme for lipid species (AEE) - Apical early endosome (ARE) - apical recycling endosome (BRE) - basolateral recycling endosome and (BEE) - basolateral early endosome.

Lipids are the main constituents within all plasma membranes, and consist of phosphoinosities, glycosphingolipids, sterols, and phosphatidylserine among others. Phosphoinositides (PIs) consist of less than 1% of the lipids within the cellular membrane, yet have been implicated in a wide variety of cellular processes, including signaling, cytoskeletal and adhesion dynamics, protein complex formation, and cell division (van Meer et al., 2008; Balla, 2013). A growing body of literature suggests that PIs and their metabolism play a crucial role in the process of cellular polarity formation. PIs undergo reversible phosphorylation and dephosphorylation by cellular kinase and phophatases, generating a number of biologically active PI species (Figure 2). Phosphorylation occurs on hydroxyl groups on the inositol ring at three positions (3,4, or 5) to produce the seven known differentially phophorylated species that exist within cells. Phosphorylated PIs originate from the ER (Figure 3A) and subsequent enzymatic reactions drive interconversion between all species. Of the seven species, here we focus on five PI species known to be involved in the generation and maintenance of epithelial polarity; PI(4)P, PI(3)P, PI(3,4)P2, PI(4,5)P2, and PI(3,4,5)P3. Enzymes regulating PI phosphorylation can be found localized to specific cellular membranes allowing for membrane, and therefore organelle, specific generation of compartmentalized pools of PI species. Spatiotemporally regulated production of PIs by specific kinases (e.g., PI4kinase and PI3kinase) or phosphatases (e.g., PTEN) allows for targeting of specific proteins to destinations within the cell. Regulation of membrane PI identity, therefore, has been proposed as a primary driving factor in polarity generation. Evidence for this as outlined in the next sections comes from a number of studies that show that loss of enzymes involved in PI phosphorylation status results in a loss of normal apico-basolateral polarity.

In contrast to the phosphoinositides, there are relatively few studies of the involvement of glycosphingolipids, cholesterol, and phosphatidylserine in the regulation of cellular polarity. Glycosphingolipids (GSLs) and cholesterol provide membrane structure by forming membrane nanodomains known as lipid rafts (Lingwood and Simons, 2010), and GSLs are enriched in specific membrane domains in organelles and vesicles along the protein secretory pathway (Zurzolo and Simons, 2016). Phosphatidylserine (PS), another key component of the cell membrane, is involved in several signaling pathways. The exposure of PS on the external leaflet of the plasma membrane is a hallmark of apoptosis, though PS has a variety of other functions in the cell (Shlomovitz et al., 2019). One role of PS that potentially impacts cell polarity is its involvement in maintenance of plasma membrane pools of PIs. In the next sections we describe in more detail the role of membrane lipids, focusing on phosphorylated PI species and important regulatory proteins involved in directing their production and localization.

## Phosphoinositides

PIs represent a minor component of plasma membranes yet control a vast array of diverse cellular functions through their recruitment of protein effectors and interaction with various PI binding domains. PIs modulate several processes including actin polymerization, growth and survival signaling and vesicular trafficking (Balla, 2013; Skwarek and Boulianne, 2009; Sasaki et al., 2009; Di Paolo and De Camilli, 2006; Burke, 2018). PIs are enriched in various membranes in unpolarized and polarized cells, with modulation of their distribution dependent on the location of specific PI kinase and phosphatase enzymes, and vesicular trafficking (Balla, 2013). (Figure 3). PI distribution within the plasma membrane changes through the process of polarity development (Figures 3A–C). The initial sources of a given PI species are highly diverse and context specific given the multiple interconversion pathways that exist (Figure 2). All phosphorylated PIs (PIPs) can originate from non-phosphorylated PIs within the Golgi following initial conversion to PI(4)P by PI4kinase (PI4K) (De Matteis et al., 2013; D'Angelo et al., 2008). PI(4)P and PI(4,5)P2 are concentrated along exocytic pathways (Ketel et al., 2016) (i.e. ER, Golgi, sorting/recycling endosomes, exocytic vesicles) as well as the plasma membranes of both polarized and non-polarized cells. PI(3)P is generated in the ER and can be found in various endosomal (Ketel et al., 2016) and autophagy-related compartments, with PI(3,4)P2 found in both endosomes and plasma membrane (Román-Fernández et al., 2018) and PI(3,4,5)P largely thought to exist on plasma membranes (Gassama-Diagne et al., 2006; van Meer et al., 2008) (Figure 3A).

The plasma membrane distribution of PIs changes considerably during the process of epithelial cell polarization. In the non-polarized cell state, PI(4,5)P2 and PI(3,4,5)P3 are thought to be equally distributed with areas enriched with PI(3,4)P2 (Figure 3A). As polarization is initiated, the PI species are initially inverted with PI(3,4,5)P3 sequestered at the cell-cell contacts and the remaining membrane containing PI(4,5)P2 (Martin-Belmonte et al., 2007) and PI(3,4)P2 (Román-Fernández et al., 2018). As polarization progresses the formation of the apical membrane initiation site (AMIS) leads to the concentration of possibly both PI(3,4)P2 (Román-Fernández et al., 2018) and PI(4,5)P2 (Martin-Belmonte et al., 2007) at the site of the eventual apical membrane, in the midst of pools of PI(3,4,5)P3. Finally, in the terminally polarizing cell, the AMIS expands to form the apical membrane containing PI(4,5)P2 and PI(3,4)P2, with PI(3,4,5)P3 now enriching the basolateral membrane (Martin-Belmonte et al., 2007; Bryant and Mostov, 2008; Román-Fernández et al., 2018) (Figure 3B).

The process of PI species interchange appears to be controlled by the membrane specific localization and trafficking of PI-kinases and phosphatases (Figure 3C) within the cell as well as chaperone proteins important for this localization. For example, the phosphatases PTEN and SHIP1 are generally located at the developing apical membrane, whereas SHIP2 and PI3K are found basolaterally (Román-Fernández et al., 2018). In the following sections we discuss the generation and specific localization of each subclass of PIs and some of the proteins that are thought to be important for their localization and/or function.

### Phosphoinositide-4-Phosphate

Phosphoinositide-4-phosphate (PI(4)P) is the most abundant of the monophosphorylated PI species in the cell. PI(4)P can be phosphorylated by both PI-3 kinases and PI-5 kinases, and is often considered an intermediate in the biosynthesis of both PI(3,4)P2 and PI(4,5)P2. However, recent studies have shown that PI(4)P itself may be important for cellular function independently of its role in generating downstream PIPs species (Zewe et al., 2020; Pemberton et al., 2020). PI(4)P is synthesized from the precursor PI by class II PI-4 kinases (PI4KIIα and PI4KIIβ) and class III PI-4 kinases (PI4KIIIα and PI4KIIIβ) (Figure 2). Class II and class III PI-4 kinases exhibit slightly different membrane distribution within cells. The Class II PI-4 kinases, PI4KIIα and PI4KIIβ are found in the Golgi, trans Golgi network (TGN), plasma membrane, and endosomes, with PI4KIIβ also in the ER. Within the Golgi and the ER, PI(4)P is necessary for the recruitment of AP-1A required for sorting to polarized membranes (Wang et al., 2003; Heldwein et al., 2004).

Class III PI-4 kinases exhibit a distinct distribution, with PI4KIIIα found in the plasma membrane, ER and nucleus and PI4KIIIβ localized to the Golgi, TGN, nucleus, endosomes and exocytic vesicles (De Matteis et al., 2013; D'Angelo et al., 2008) (Figure 3c). Class III PI-4 kinases appear to have distinct functions in the organization of the apical and basolateral domains. PI4KIIIα and PI4KIIIβ and subsequently PI(4)P generation are postulated to be involved in Rab11a driven vesicular trafficking of membrane proteins to the apical membrane (Bruns et al., 2002; Yan et al., 2011; Tan et al., 2014). PI4KIIIα in particular may be involved in regulating of PI(4)P at the plasma membrane (Baskin et al., 2016).

PI4KIIIα is thought to be localized and function in membranes by assembly within a multi-protein complex, which includes the putative chaperone proteins, TTC7A/B, and Fam126, and tethered to the plasma membrane by the anchor protein Efr3 (Baird et al., 2008; Dornan et al., 2018) In yeast, the PI4KIIIα homolog Stt4p is found on the plasma membrane where it regulates actin and cytoskeleton organization (Audhya et al., 2000). In oocytes the loss of PI4KIIIα results in malformed microvilli, and in the intestine, human loss of function mutations in TTC7A result in abnormal apico-basolateral polarity (Bigorgne et al., 2014) and crypt glands with multiple lumens (Jardine et al., 2019) conceivably due to loss of either PI4KIIIα localization or function. There is increasing evidence that this protein complex may play an important role in intestinal morphogenesis, apical basolateral polarity and lumen formation (Jardine et al., 2019; Avitzur et al., 2014). Multiple different patient mutations that alter TTC7A protein levels or function appear to manifest as a variety of architectural defects including the formation of multiple intestinal atresias as well as severely abnormal intestinal crypt gland lumen formation. It remains unclear whether the observed alterations in apico-basolateral polarity are due to a direct loss of PI(4)P (Avitzur et al., 2014) within a cell, or secondary to the subsequent loss of downstream phosphorylated PI species. Historically, there has been a general assumption that PI(4)P primarily acts as a precursor for the synthesis of PI(4,5)P2 (Hammond et al., 2012). However, PI(4)P is also a major a precursor for PI(3,4)P2 within a cell **(** Figure 2). Emerging studies suggest that PI(3,4)P2 is critical for driving transcytotic pathways from the basolateral surface to the apical membrane during the process of polarization (Román-Fernández et al., 2018) and loss of PI(3,4)P2 prevents normal lumen formation (Román-Fernández et al., 2018). As with other PIs, the downstream mechanisms by which the loss of the TTC7A-PI4KIIIα complex and PI(4)P may lead to abnormal polarity likely occurs via alteration in the binding and localization of polarity complexes at the plasma membrane. PI(4)P, like many other PIs, serves as a scaffold/anchorage point at the plasma membrane for protein complexes. In a polarized cell, Par1 and the Scribble complex are often seen at PI(4)P rich domains (Dong et al., 2015) and plasma membrane targeting of Lgl is partly dependent on its interaction with PI(4)P at the basolateral plasma membrane (Krahn et al., 2010; Hammond and Hong, 2018). Further studies are needed to determine exactly how loss of TTC7A function alters PI4KIIIα in the intestinal epithelium and whether and critically *where* in the cell this may impact PI(4)P, PI(3,4)P2 or PI(4,5)P2 biogenesis and subsequent polarity complex formation.

### Phosphoinositide-3-Phosphate

The other main monophosphorylated PI that may be involved in cell polarity is PI(3)P. PI(3)P is generated by Class I and III PI3-kinases or by dephosphorylation of PI(3,4)P2 by INPP4A/B. PI(3)P has been primarily studied for its role in modulating autophagy (Nascimbeni et al., 2017). There are relatively few studies that have studied PI(3)P during polarity. One potential intersection between PI(3)P and polarity is via endocytic trafficking and recruitment of proteins such as early endosome antigen 1 (EEA1). EEA1 is a Rab5A effector protein required for sorting at the early endosome. Endosomal localization of EEA1 by interactions with PI(3)P allows Rab5 to act in concert with PI(3)P to regulate polarized membrane trafficking (Mayinger, 2012), including recycling or sorting of apical membrane proteins to their correct location. While PI(3)P has been well-studied for its role in endocytosis and endosomal dynamics, it is unclear from the current literature if PI(3)P levels within the cell can modulate polarity by aiding the formation of PI(3,4)P2 and PI(3,4,5)P3. In cyst formation models, PI(3)P positive vesicles appear to accumulate below the forming AMIS (Román-Fernández et al., 2018). Despite the localization of these endosomes, these PI(3)P positive vesicles do not deliver proteins to the AMIS (Román-Fernández et al., 2018), although it is possible that apical cargo may potentially pass through PI(3)P positive vesicles en route to the forming AMIS.

### Phosphoinositide-4,5-Phosphate

PI(4,5)P2 is by far the most well studied PI species in epithelial cell polarization (Roignot et al., 2013). PI(4,5)P2 is involved in a wide variety of cellular functions; it serves as a substrate for phospholipase C cleavage in G-protein coupled signaling cascades, as a precursor for PI(3,4,5)P3 by Class I PI3-kinases, and as a binding molecule for the recruitment of many membrane associated proteins (Balla, 2013). A number of studies have shown that PI(4,5)P2 segregates at the forming apical membrane early in polarization (Martin-Belmonte et al., 2007; Román-Fernández et al., 2018). As cell polarization continues PI(4,5)P2 recruits several polarity complex proteins, via direct interactions, towards the forming apical membrane (Balla, 2013). PI(4,5)P2 promotes localization of the Par3 and Par6 proteins apically through interactions with the PDZ domains of the proteins, as well as facilitating the apical localization of ezrin, radixin and moesin through direct binding of their FERM domains (Michie et al., 2019). Loss of PI(4,5)P2 or loss of binding of these complexes to PI(4,5)P2 in a polarizing cell impairs epithelial polarity. Segregation of PI(4,5)P at the apical domain can occur either by phosphorylation of PI(4)P by PI-5 kinases, or dephosphorylation of PI(3,4,5)P3 by the phosphatase PTEN (Martin-Belmonte et al., 2007). Interestingly, at the forming apical membrane the action of PTEN itself facilitates protein localization. Loss of PTEN activity causes ectopic PI(4,5)P2 localization, preventing PI(4,5)P2 binding to Annexin and subsequent recruitment and activation of Cdc42 and aPKC (Martin-Belmonte et al., 2007). Regulation of PTEN can also occur by Rho-kinase mediated phosphorylation (Sanchez et al., 2005), and both the Rac1 and Rho GTPases activate PI-5-kinase (Weernink et al., 2000) thus providing additional mechanisms of promoting PI(4,5)P2 production. Rho in particular has been proposed to be involved in the production of PI(4,5)P2 during polarity, following the finding that use of the Rho-kinase inhibitor Y27632 can potentially remedy altered polarity in certain disease states (Bigorgne et al., 2014).

Although multiple studies have reported the segregation of PI(4,5)P2 at the apical membrane, during its formation, opinions about its relative importance and position in the process of polarity have varied. Early studies in cyst models proposed PI(4,5)P2 as the critical PI species during *de novo* polarity generation, with later studies using the same models amending this hypothesis (Martin-Belmonte et al., 2007; Román-Fernández et al., 2018). Further, conflicting results in a different models or tissues have also suggested that the role of PI(4,5)P2 may be context specific. The bulk of the data supporting a pre-eminent role for PI(4,5)P2 in polarization has come from kidney (MDCK) cysts (Gassama-Diagne et al., 2006; Martin-Belmonte et al., 2007; Román-Fernández et al., 2018), with similar findings in colonic T84 cysts (Martin-Belmonte et al., 2007). However, in contrast to MDCK cyst models (Martin-Belmonte et al., 2007), the loss of apical PTEN localization resulted in the expansion of the apical membrane domain in the intestinal cell line LS174T:W4 – an inducible single cell polarity model forming microvilli-like apical plasma membranes (Gloerich et al., 2012; Bruurs et al., 2018), rather than the suppression of the apical domain as previously seen (Martin-Belmonte et al., 2007). Further, *in vivo* studies of the rat pancreas showed that PI(4,5)P2 is only enriched at the gap junctions with an equivalent distribution in the apical and basolateral domains (van Zeijl et al., 2007). More work remains fully understand the role of PI(4,5)P2 in polarization, and understand where PI(4,5)P2 fits into the emerging model that the other major PIP2 species - PI(3,4)P2 - may in fact be an earlier and therefore more important factor in apical membrane specification (Román-Fernández et al., 2018).

### Phosphoinositide-3,4-Phosphate

Despite the lack of clear signaling functions in the cell, PI(3,4)P2 is emerging as the potential key PI species in the establishment and maintenance of apical and basolateral surfaces. Historically, a role for PI(3,4)P2 has been implicated in cell motility, and endocytosis (Balla, 2013). In polarized cells, PI(3,4)P2 was found to have a prominent role in the maintenance of basolateral polarity complexes by SHIP2 dependent dephosphorylation of PI(3,4,5)P3 (Awad et al., 2013). The localization of SHIP2 at cell-ECM contact sites provide further impetus for the idea that generation of PI(3,4)P2 was mostly important at basolateral membranes (Awad et al., 2013).

However in recent and fascinating studies in epithelial cysts, a primary role for PI(3,4)P2 in formation of the apical membrane has emerged (Román-Fernández et al., 2018). These studies showed that PI(3,4)P2 is also found together with Rab11a sorting endosomes at the forming AMIS and later at mature apical membranes (Román-Fernández et al., 2018). It is thought that SHIP1 (PI(3,4,5)P3 → PI(3,4)P2) may play a critical role in initial polarization, but not in maintenance of the membrane. During epithelial polarization, SHIP1 recruits Par3 localization apically and maintains apical pools of PI(3,4)P2. Disruption of SHIP1 during cyst polarization consequently was found to alter apical membrane trafficking and lumen formation. However, it appears that SHIP1 and subsequent PI(3,4)P2 generation is not involved in apical membrane maintenance in fully polarized cells, as there was no effect of loss of SHIP1 in cysts once lumens had already formed (Román-Fernández et al., 2018).

PI(4P) to PI(3,4)P2 biosynthesis may also play a role in polarity generation and lumen formation via the class II PI3kinase isoforms PIK3C2A, PIK3C2B and PIK3C2G. Loss of either PIK3C2A or PIK3C2B results in a loss of *de novo* polarity formation, and causes the presence in cysts of multiple lumens (Román-Fernández et al., 2018). Conversely, the loss of the PIK3C2G isoform appears to promote lumen formation (Román-Fernández et al., 2018). These divergent effects between PI3K isoforms may be in part explained by their differential cellular localization and, therefore, role during early polarity formation. PIK3C2A is closely associated with Rab11a vesicles throughout polarization, whereas, PI3KC2B is initially seen in cells at the pre-apical membrane, but is trafficked to the basolateral surface in Rab11a positive vesicles (Román-Fernández et al., 2018). How PIK3C2G is involved in polarity and lumen formation remains unclear. Nevertheless, there appears to be dynamic regulation of PI(3,4)P2 by different Class II PI3K isoforms, and the preferential localization of these enzymes at different membranes may be critical to lumenogenesis and the sorting of apical and basolateral determinants to the correct membranes.

The role of PI(3,4)P2 in localizing polarity proteins appears to differ from PI(4,5)P2. Loss of PI(3,4)P2 generation does not appear to affect aPKC/Par6/Cdc42 or Annexin2 localization in contrast to loss of PI(4,5)P2 (Román-Fernández et al., 2018). However other PI(3,4)P2 -protein interactions do appear to be critical with the finding that PI(3,4)P2 is required for binding with Syntaxin 9 (SNX9), with loss of this interaction resulting in disrupted AMIS formation (Román-Fernández et al., 2018). Despite these intriguing results for PI(3,4)P2, the relative importance, temporal hierarchy and generalizability to other epithelial cells of the two PIP2 species in polarity generation, remains an open question and an important area for future studies. The studies thus far suggest that both PI(4,5)P2 and PI(3,4)P2 are essential, but may promote different aspects of protein sorting during apical membrane and subsequently lumen formation.

### Phosphoinositide-3,4,5-Phosphate

Numerous studies have established that, in polarized epithelial cells, PI(3,4,5)P3 is typically excluded from the apical membrane and localized at the basolateral membrane (Watton and Downward, 1999; Gassama-Diagne et al., 2006; Martin-Belmonte et al., 2007; Takahama et al., 2008; Román-Fernández et al., 2018). PI(3,4,5)P3 displays a dynamic localization during polarization. In an unpolarized cell, PI(3,4,5)P3 is somewhat evenly distributed along the cellular membrane. When a cell undergoes division, initiating the process of polarization, PI(3,4,5)P3 is typically enriched the plasma membrane at cell-cell contacts that ultimately become the developing apical membrane. (Gassama-Diagne et al., 2006; Martin-Belmonte et al., 2007; Román-Fernández et al., 2018). After this initial cell division with ‘inverted’ PI polarity, as described above, apical accumulation of PI(4,5)P2 occurs by apical PTEN recruitment (Martin-Belmonte et al., 2007) and PI(3,4)P2 appears on vesicles that are trafficked from the basolateral surface to the forming apical membrane (Román-Fernández et al., 2018). How basolateral PI(3,4,5)P3 is maintained during the process of polarity and especially after the formation of the apical membrane remains unclear. Current models largely attribute the presence of basolateral PI(3,4,5)P3 as a secondary consequence of reorganization of PI(4,5)P2 and PI(3,4)P2 to the apical membrane. Maintenance of PI(3,4,5)P3 levels in cells is dependent on Class I PI-3 kinases and PI-5 kinases **(** Figure 2) (Sasaki et al., 2009; Balla, 2013; Burke, 2018). In epithelial cells, the localization of Class I PI-3 kinases is dynamically regulated, and is thought to occur in part by lateral and basolateral membrane proteins such as Dlg, *β*-1 integrin, and E-cadherin (Pece et al., 1999). It is postulated that PI3K is recruited and subsequently activated by Dlg, as E-cadherin cell-cell adhesions occur. This idea was further supported by studies showing that E-cadherin and laminin in the basolateral membrane provide a pool of active PI-3 kinase during polarization (Pece et al., 1999; Xu et al., 2010), whereas the apical Crumbs complex inhibits PI3K activity, allowing for localized effects of PI3K at the basolateral surface. Importantly, PI(3,4,5)P3 is needed for localization of AP-1B in recycling endosomes and basolateral sorting of AP-1B–dependent cargos (Fields et al., 2010), suggesting a secondary mechanism by which endosomal trafficking drives PIP3 localization in the polarized epithelium. Transferrin positive recycling endosomes contain PI(3,4,5)P3 colocalized with AP-1B (Fields et al., 2010). PI4P 5-kinase (PIPKIγ-90) colocalizes with TfnR in recycling endosomes and this colocalization depends on AP-1B expression (Ling et al., 2007; Fields et al., 2010).

Overall, the current evidence suggests that the role of PI(3,4,5)P3 in *de novo* polarization is largely secondary to the PIP2 species, although whether this is the case for maintenance of existing polarity is unclear, and more studies are needed to clarify the mechanisms that restrict PI(3,4,5)P3 to the basolateral domain.

## Glycosphingolipids/Lipid Rafts and Phosphatidylserine

Glycosphingolipids (GSL) are a class of glycolipids consisting of a ceramide backbone bound to a complex glycan headgroup. GSL synthesis is complex, with lipid synthesis originating in the ER with glycosylation (attachment of the glycan) occurring within the Golgi (D'Angelo et al., 2013), resulting in several GSL species (e.g., GM1, GM3, GB3, GT1 etc). The function of GSLs within cells are wide-ranging, and include modulation of membrane-protein function and cell-cell communication (D'Angelo et al., 2013). Examples of direct GSL-protein interactions which modulate function, include the interaction between GM3 and EGFR, or interactions with structural membrane proteins such as integrins (D'Angelo et al., 2013). It is postulated that glycosphingolipids are responsible for forming functional domains with the epithelial apical membrane and serve as a protective barrier for the epithelial monolayer. In keeping with this role, the apical plasma membrane domain of epithelial cells is enriched in GSLs. This apical membrane enrichment of GSLs may impact cell polarization by potential GSL-protein interactions. GSL sorting from the TGN to the apical membrane may drive the localization of several apically functioning proteins (Brown et al., 1989; Lisanti et al., 1989; Mishra et al., 2010; Zurzolo and Simons, 2016). Previous work has shown that as a cell begins to polarize that the acyl chain length, hydroxylation status and glycosylation of GSLs are altered favoring GSL sequestration within the apical membrane (Nichols et al., 1987; Sampaio et al., 2011; Wattelet-Boyer et al., 2016). In addition, sequestration of GSLs may occur due in coordination with local cholesterol rich domains in the apical microvillus membrane domain of intestinal epithelial cells (Hansen et al., 2003; Kunding et al., 2010; Zurzolo and Simons, 2016). A potential role for GSLs in the genesis of the apical membrane is further validated by knockdown studies, where inhibition of glycosphingolipid synthesis can prevent normal formation of the apical membrane in kidney cells (Pescio et al., 2012), with further evidence later provided by a genetic screening studies in *C. elegans* intestine (Zhang et al., 2011). A particularly interesting observation in these studies was that restoration of GSL lipid biosynthesis is sufficient to reverse the multiple lumen phenotype induced by loss of GSLs (Zhang et al., 2011; Wattelet-Boyer et al., 2016). Although there are relatively few studies that have investigated in detail the role of GSLs in epithelial polarity formation, the data from cell lines and C.elegans suggest an underappreciated role for GSLs in the establishment and function of the apical membrane.

### Phosphatidylserine

Phosphatidylserine (PS) is a negatively charged phospholipid that displays a highly uneven distribution within cellular membranes and is essential for several cellular processes. PS biosynthesis occurs in the endoplasmic reticulum, followed by transport to the plasma membrane. Similar to PIs, phosphatidylserine serves several purposes within the cell, including modulating both protein localization and lipid homeostasis and localization (Fairn et al., 2011; Moser von Filseck et al., 2015; Sartorel et al., 2018; Lenoir et al., 2021). Phosphatidylserine is required for the localization of CDC42 and cell polarity formation in yeast (*S cerevisiae*), in part due to localized clustering of PS at the apical membrane (Fairn et al., 2011; Sartorel et al., 2018). The polarized distribution of PS is not unique to yeast, as PS is also shown to be localized to the apical plasma membrane in mammalian systems as well (Shi et al., 2007). Further work in yeast and mammalian systems show that the PI4KIIIα and its ortholog, Stt4 (Trotter et al., 1998) are essential for the proper generation and clustering of PS in the cell, while increased PS in the ER has been linked to activation of the PI(4)P phosphatase Sac1 (Sohn and Balla, 2016). The dynamic interplay between PI(4)P and PS is not unsurprising as PS transport to the plasma membrane is tightly coupled to PI(4)P-dependent transport at ER-plasma membrane contacts sites (Sohn and Balla, 2016). Given that PS is apically enriched (Shi et al., 2007), and that PI4KIIIα (Trotter et al., 1998) and PI(4)P (Sohn and Balla, 2016) are required for the proper localization, PS, PI(4)P and PI4KIIIα may all be sequestered together within the apical domain. The PI4KIIIα dependent apical enrichment of PS adds to the concept that the PI4KIIIα/TTC7A/B complex is important in polarity generation.

## Conclusion

Apical-basolateral polarity generation and lumen morphogenesis are fundamental and multifaceted processes that occur across different organs and tissues. The protein complexes involved in this process have been generally studied extensively, while the role of the lipid components of membranes remains an emerging field. Several lipid species, including most notably the PIs have been identified as crucial for the correct sequence of events that underlies polarity generation. The exact proteins-complexes and lipid species that are involved, likely varies between epithelial cell types, and tube formation mechanisms may be subtly, but importantly, different between organs such as the kidney and the intestine. In intestinal epithelia, a variety of mechanisms contribute to the distribution of PIs, GSLs, and PS in the polarizing epithelium, with evidence emerging for a central role for protein-enzyme complexes important for PI species generation such the PI4KIIIα/TTC7, PTEN/CDC42 and Rac/Rho complexes. The relative importance of individual PI species in polarity remains to be fully understood, and further work is needed to investigate this aspect. It is, however, becoming increasingly clear that vesicular trafficking events are critical early in the process of defining the apical and basolateral membrane domains, although exactly how PIs are involved remains to be fully characterized.

In summary, there is growing appreciation for the importance of membrane lipids such as the PIs, GSLs and PS in epithelial cell polarity. Future studies focusing on these membrane lipids, including overcoming the considerable technical challenges inherent in studying lipids, will help further illuminate our understanding of the process of polarization in epithelia.

## References

[B1] Alvers A. L. Ryan S. Scherz P. J. Huisken J. Bagnat M. (2014). Single Continuous Lumen Formation in the Zebrafish Gut Is Mediated by Smoothened-dependent Tissue Remodeling. Dev. Camb. Engl. 141, 1110–1119. 10.1242/dev.100313 PMC3929411 24504339

[B2] Audhya A. Foti M. Emr S. D. (2000). Distinct Roles for the Yeast Phosphatidylinositol 4-kinases, Stt4p and Pik1p, in Secretion, Cell Growth, and Organelle Membrane Dynamics. MBoC 11, 2673–2689. 10.1091/mbc.11.8.2673 10930462 PMC14948

[B3] Avitzur Y. Guo C. Mastropaolo L. A. Bahrami E. Chen H. Zhao Z. (2014). Mutations in Tetratricopeptide Repeat Domain 7A Result in a Severe Form of Very Early Onset Inflammatory Bowel Disease. Gastroenterology 146, 1028–1039. 10.1053/j.gastro.2014.01.015 24417819 PMC4002656

[B4] Awad A. Sar S. Barré R. Cariven C. Marin M. Salles J. P. (2013). SHIP2 Regulates Epithelial Cell Polarity through its Lipid Product, Which Binds to Dlg1, a Pathway Subverted by Hepatitis C Virus Core Protein. MBoC 24, 2171–2185. 10.1091/mbc.e12-08-0626 23699395 PMC3708724

[B5] Bagnat M. Cheung I. D. Mostov K. E. Stainier D. Y. R. (2007). Genetic Control of Single Lumen Formation in the Zebrafish Gut. Nat. Cell Biol. 9, 954–960. 10.1038/ncb1621 17632505

[B6] Baird D. Stefan C. Audhya A. Weys S. Emr S. D. (2008). Assembly of the PtdIns 4-kinase Stt4 Complex at the Plasma Membrane Requires Ypp1 and Efr3. J. Cell Biol. 183, 1061–1074. 10.1083/jcb.200804003 19075114 PMC2600738

[B7] Balla T. P. (2013). Tiny Lipids with Giant Impact on Cell Regulation. Physiol. Rev. 93, 119. 10.1152/physrev.00028.2012 PMC3962547 23899561

[B8] Baskin J. M. Wu X. Christiano R. Oh M. S. Schauder C. M. Gazzerro E. (2016). The Leukodystrophy Protein FAM126A (Hyccin) Regulates PtdIns(4)P Synthesis at the Plasma Membrane. Nat. Cell Biol. 18, 132–138. 10.1038/ncb3271 26571211 PMC4689616

[B9] Bigorgne A. E. Farin H. F. Lemoine R. Mahlaoui N. Lambert N. Gil M. (2014). TTC7A Mutations Disrupt Intestinal Epithelial Apicobasal Polarity. J. Clin. Invest. 124, 328–337. 10.1172/jci71471 24292712 PMC3871247

[B10] Blasky A. J. Mangan A. Prekeris R. (2015). Polarized Protein Transport and Lumen Formation during Epithelial Tissue Morphogenesis. Annu. Rev. Cell Dev. Biol. 31, 575–591. 10.1146/annurev-cellbio-100814-125323 26359775 PMC4927002

[B11] Bonifacino J. S. Traub L. M. (2003). Signals for Sorting of Transmembrane Proteins to Endosomes and Lysosomes. Annu. Rev. Biochem. 72, 395–447. 10.1146/annurev.biochem.72.121801.161800 12651740

[B12] Brown D. A. Crise B. Rose J. K. (1989). Mechanism of Membrane Anchoring Affects Polarized Expression of Two Proteins in MDCK Cells. Science 245, 1499–1501. 10.1126/science.2571189 2571189

[B13] Bruns J. R. Ellis M. A. Jeromin A. Weisz O. A. (2002). Multiple Roles for Phosphatidylinositol 4-kinase in Biosynthetic Transport in Polarized Madin-Darby Canine Kidney Cells. J. Biol. Chem. 277, 2012–2018. 10.1074/jbc.M108571200 11704666

[B14] Bruurs L. J. M. van der Net M. C. Zwakenberg S. Rosendahl Huber A. K. M. Post A. Zwartkruis F. J. (2018). The Phosphatase PTPL1 Is Required for PTEN-Mediated Regulation of Apical Membrane Size. Mol. Cell. Biol. 38, e00102–18. 10.1128/MCB.00102-18 29581186 PMC5974425

[B15] Bryant D. M. Datta A. Rodríguez-Fraticelli A. E. Peränen J. Martín-Belmonte F. Mostov K. E. (2010). A Molecular Network for De Novo Generation of the Apical Surface and Lumen. Nat. Cell Biol. 12, 1035–1045. 10.1038/ncb2106 20890297 PMC2975675

[B16] Bryant D. M. Mostov K. E. (2008). From Cells to Organs: Building Polarized Tissue. Nat. Rev. Mol. Cell Biol. 9, 887–901. 10.1038/nrm2523 18946477 PMC2921794

[B17] Bryant D. M. Roignot J. Datta A. Overeem A. W. Kim M. Yu W. (2014). A Molecular Switch for the Orientation of Epithelial Cell Polarization. Dev. Cell 31, 171–187. 10.1016/j.devcel.2014.08.027 25307480 PMC4248238

[B18] Buckley A. Turner J. R. (2018). Cell Biology of Tight Junction Barrier Regulation and Mucosal Disease. Cold Spring Harb. Perspect. Biol. 10, a029314. 10.1101/cshperspect.a029314 28507021 PMC5749156

[B19] Burke J. E. (2018). Structural Basis for Regulation of Phosphoinositide Kinases and Their Involvement in Human Disease. Mol. Cell 71, 653–673. 10.1016/j.molcel.2018.08.005 30193094

[B20] Chartier F. J.-M. Hardy É. J.-L. Laprise P. (2011). Crumbs Controls Epithelial Integrity by Inhibiting Rac1 and PI3K. J. Cell Sci. 124, 3393–3398. 10.1242/jcs.092601 21984807

[B21] D'Angelo G. Capasso S. Sticco L. Russo D. (2013). Glycosphingolipids: Synthesis and Functions. FEBS J. 280, 6338–6353. 10.1111/febs.12559 24165035

[B22] D'Angelo G. Vicinanza M. Di Campli A. De Matteis M. A. (2008). The Multiple Roles of PtdIns(4)P -- Not Just the Precursor of PtdIns(4,5)P2. J. Cell Sci. 121, 1955–1963. 10.1242/jcs.023630 18525025

[B23] De Matteis M. A. Wilson C. D'Angelo G. (2013). Phosphatidylinositol-4-phosphate: the Golgi and beyond. BioEssays 35, 612–622. 10.1002/bies.201200180 23712958

[B24] Di Paolo G. De Camilli P. (2006). Phosphoinositides in Cell Regulation and Membrane Dynamics. Nature 443, 651–657. 10.1038/nature05185 17035995

[B25] Dong W. Zhang X. Liu W. Chen Y.-j. Huang J. Austin E. (2015). A Conserved Polybasic Domain Mediates Plasma Membrane Targeting of Lgl and its Regulation by Hypoxia. J. Cell Biol. 211, 273–286. 10.1083/jcb.201503067 26483556 PMC4621827

[B26] Dornan G. L. Dalwadi U. Hamelin D. J. Hoffmann R. M. Yip C. K. Burke J. E. (2018). Probing the Architecture, Dynamics, and Inhibition of the PI4KIIIα/TTC7/FAM126 Complex. J. Mol. Biol. 430, 3129–3142. 10.1016/j.jmb.2018.07.020 30031006

[B27] Fairn G. D. Hermansson M. Somerharju P. Grinstein S. (2011). Phosphatidylserine Is Polarized and Required for Proper Cdc42 Localization and for Development of Cell Polarity. Nat. Cell Biol. 13, 1424–1430. 10.1038/ncb2351 21964439

[B28] Fehon R. G. McClatchey A. I. Bretscher A. (2010). Organizing the Cell Cortex: The Role of ERM Proteins. Nat. Rev. Mol. Cell Biol. 11, 276–287. 10.1038/nrm2866 20308985 PMC2871950

[B29] Ferrari A. Veligodskiy A. Berge U. Lucas M. S. Kroschewski R. (2008). ROCK-mediated Contractility, Tight Junctions and Channels Contribute to the Conversion of a Preapical Patch into Apical Surface during Isochoric Lumen Initiation. J. Cell Sci. 121, 3649–3663. 10.1242/jcs.018648 18946028

[B30] Fields I. C. King S. M. Shteyn E. Kang R. S. Fölsch H. (2010). Phosphatidylinositol 3,4,5-trisphosphate Localization in Recycling Endosomes Is Necessary for AP-1b-dependent Sorting in Polarized Epithelial Cells. MBoC 21, 95–105. 10.1091/mbc.e09-01-0036 19864464 PMC2801725

[B31] Fölsch H. Pypaert M. Maday S. Pelletier L. Mellman I. (2003). The AP-1A and AP-1B Clathrin Adaptor Complexes Define Biochemically and Functionally Distinct Membrane Domains. J. Cell Biol. 163, 351–362. 10.1083/jcb.200309020 14581457 PMC2173537

[B32] Fölsch H. (2005). The Building Blocks for Basolateral Vesicles in Polarized Epithelial Cells. Trends Cell Biol. 15, 222–228. 10.1016/j.tcb.2005.02.006 15817379

[B33] Gálvez-Santisteban M. (2012). Synaptotagmin-like Proteins Control the Formation of a Single Apical Membrane Domain in Epithelial Cells. Nat. Cell Biol. 14, 838–849. 10.1038/ncb2541 22820376 PMC3433678

[B34] Garcia-Castillo M. D. Chinnapen D. J.-F. Lencer W. I. (2017). Membrane Transport across Polarized Epithelia. Cold Spring Harb. Perspect. Biol. 9, a027912. 10.1101/cshperspect.a027912 28213463 PMC5585844

[B35] Gassama-Diagne A. Yu W. ter Beest M. Martin-Belmonte F. Kierbel A. Engel J. (2006). Phosphatidylinositol-3,4,5-trisphosphate Regulates the Formation of the Basolateral Plasma Membrane in Epithelial Cells. Nat. Cell Biol. 8, 963–970. 10.1038/ncb1461 16921364

[B36] Gloerich M. ten Klooster J. P. Vliem M. J. Koorman T. Zwartkruis F. J. Clevers H. (2012). Rap2A Links Intestinal Cell Polarity to Brush Border Formation. Nat. Cell Biol. 14, 793–801. 10.1038/ncb2537 22797597

[B37] Gravotta D. Carvajal-Gonzalez J. M. Mattera R. Deborde S. Banfelder J. R. Bonifacino J. S. (2012). The Clathrin Adaptor AP-1A Mediates Basolateral Polarity. Dev. Cell 22, 811–823. 10.1016/j.devcel.2012.02.004 22516199 PMC3690600

[B38] Gravotta D. Perez Bay A. Jonker C. T. H. Zager P. J. Benedicto I. Schreiner R. (2019). Clathrin and Clathrin Adaptor AP-1 Control Apical Trafficking of Megalin in the Biosynthetic and Recycling Routes. MBoC 30, 1716–1728. 10.1091/mbc.e18-12-0811 31091172 PMC6727755

[B39] Hammond G. R. Hong Y. (2018). Phosphoinositides and Membrane Targeting in Cell Polarity. Cold Spring Harb. Perspect. Biol. 10, a027938. 10.1101/cshperspect.a027938 28264819 PMC5793754

[B40] Hammond G. R. V. Fischer M. J. Anderson K. E. Holdich J. Koteci A. Balla T. (2012). PI4P and PI(4,5)P 2 Are Essential but Independent Lipid Determinants of Membrane Identity. Science 337, 727–730. 10.1126/science.1222483 22722250 PMC3646512

[B41] Hansen G. H. Pedersen J. Niels-Christiansen L.-L. Immerdal L. Danielsen E. M. (2003). Deep-apical Tubules: Dynamic Lipid-Raft Microdomains in the Brush-Border Region of Enterocytes. Biochem. J. 373, 125–132. 10.1042/bj20030235 12689332 PMC1223483

[B42] Heldwein E. E. Macia E. Wang J. Yin H. L. Kirchhausen T. Harrison S. C. (2004). Crystal Structure of the Clathrin Adaptor Protein 1 Core. Proc. Natl. Acad. Sci. U.S.A. 101, 14108–14113. 10.1073/pnas.0406102101 15377783 PMC521094

[B43] Iden S. Collard J. G. (2008). Crosstalk between Small GTPases and Polarity Proteins in Cell Polarization. Nat. Rev. Mol. Cell Biol. 9, 846–859. 10.1038/nrm2521 18946474

[B44] Jardine S. Dhingani N. Muise A. M. (2019). TTC7A: Steward of Intestinal Health. Cell. Mol. Gastroenterology Hepatology 7, 555–570. 10.1016/j.jcmgh.2018.12.001 PMC6406079 30553809

[B45] Jewett C. E. Prekeris R. (2018). Insane in the Apical Membrane: Trafficking Events Mediating Apicobasal Epithelial Polarity during Tube Morphogenesis. Traffic 19, 666–678. 10.1111/tra.12579 PMC6239989 29766620

[B46] Ketel K. Krauss M. Nicot A.-S. Puchkov D. Wieffer M. Müller R. (2016). A Phosphoinositide Conversion Mechanism for Exit from Endosomes. Nature 529, 408–412. 10.1038/nature16516 26760201

[B47] Krahn M. P. Klopfenstein D. R. Fischer N. Wodarz A. (2010). Membrane Targeting of Bazooka/PAR-3 Is Mediated by Direct Binding to Phosphoinositide Lipids. Curr. Biol. 20, 636–642. 10.1016/j.cub.2010.01.065 20303268

[B48] Kunding A. H. Christensen S. M. Danielsen E. M. Hansen G. H. (2010). Domains of Increased Thickness in Microvillar Membranes of the Small Intestinal Enterocyte. Mol. Membr. Biol. 27, 170–177. 10.3109/09687688.2010.494625 20540667

[B49] Lenoir G. D’Ambrosio J. M. Dieudonné T. Čopič A. (2021). Transport Pathways that Contribute to the Cellular Distribution of Phosphatidylserine. Front. Cell Dev. Biol. 9, 2412. 10.3389/fcell.2021.737907 PMC8440936 34540851

[B50] Lin D. Edwards A. S. Fawcett J. P. Mbamalu G. Scott J. D. Pawson T. (2000). A Mammalian PAR-3-PAR-6 Complex Implicated in Cdc42/Rac1 and aPKC Signalling and Cell Polarity. Nat. Cell Biol. 2, 540–547. 10.1038/35019582 10934475

[B51] Ling K. Bairstow S. F. Carbonara C. Turbin D. A. Huntsman D. G. Anderson R. A. (2007). Type Iγ Phosphatidylinositol Phosphate Kinase Modulates Adherens Junction and E-Cadherin Trafficking via a Direct Interaction with μ1B Adaptin. J. Cell Biol. 176, 343–353. 10.1083/jcb.200606023 17261850 PMC2063960

[B52] Lingwood D. Simons K. (2010). Lipid Rafts as a Membrane-Organizing Principle. Science 327, 46–50. 10.1126/science.1174621 20044567

[B53] Lisanti M. P. Caras I. W. Davitz M. A. Rodriguez-Boulan E. (1989). A Glycophospholipid Membrane Anchor Acts as an Apical Targeting Signal in Polarized Epithelial Cells. J. Cell Biol. 109, 2145–2156. 10.1083/jcb.109.5.2145 2478564 PMC2115867

[B54] Macara I. G. (2004). Par Proteins: Partners in Polarization. Curr. Biol. 14, R160–R162. 10.1016/j.cub.2004.01.048 15027470

[B55] Madara J. L. Neutra M. R. Trier J. S. (1981). Junctional Complexes in Fetal Rat Small Intestine during Morphogenesis. Dev. Biol. 86, 170–178. 10.1016/0012-1606(81)90327-4 7286391

[B56] Martin-Belmonte F. Gassama A. Datta A. Yu W. Rescher U. Gerke V. (2007). PTEN-mediated Apical Segregation of Phosphoinositides Controls Epithelial Morphogenesis through Cdc42. Cell 128, 383–397. 10.1016/j.cell.2006.11.051 17254974 PMC1865103

[B57] Mathan M. Moxey P. C. Trier J. S. (1976). Morphogenesis of Fetal Rat Duodenal Villi. Am. J. Anat. 146, 73–92. 10.1002/aja.1001460104 937208

[B58] Mayinger P. (2012). Phosphoinositides and Vesicular Membrane Traffic. Biochimica Biophysica Acta (BBA) - Mol. Cell Biol. Lipids 1821, 1104–1113. 10.1016/j.bbalip.2012.01.002 PMC3340507 22281700

[B59] McClatchey A. I. (2012). ERM Proteins. Curr. Biol. 22, R784–R785. 10.1016/j.cub.2012.07.057 23017986

[B60] Michie K. A. Bermeister A. Robertson N. O. Goodchild S. C. Curmi P. M. G. (2019). Two Sides of the Coin: Ezrin/Radixin/Moesin and Merlin Control Membrane Structure and Contact Inhibition. Ijms 20, 1996. 10.3390/ijms20081996 PMC6515277 31018575

[B61] Mishra R. Grzybek M. Niki T. Hirashima M. Simons K. (2010). Galectin-9 Trafficking Regulates Apical-Basal Polarity in Madin-Darby Canine Kidney Epithelial Cells. Proc. Natl. Acad. Sci. U.S.A. 107, 17633–17638. 10.1073/pnas.1012424107 20861448 PMC2955135

[B62] Moreno-Roman P. Su Y.-H. Galenza A. Acosta-Alvarez L. Debec A. Guichet A. (2021). Progenitor Cell Integration into a Barrier Epithelium during Adult Organ Turnover. 457819, 10.1101/2021.09.19.457819

[B63] Moser von Filseck J. Čopič A. Delfosse V. Vanni S. Jackson C. L. Bourguet W. (2015). Phosphatidylserine Transport by ORP/Osh Proteins Is Driven by Phosphatidylinositol 4-phosphate. Science 349, 432–436. 10.1126/science.aab1346 26206936

[B64] Nascimbeni A. C. Codogno P. Morel E. (2017). Phosphatidylinositol‐3‐phosphate in the Regulation of Autophagy Membrane Dynamics. FEBS J. 284, 1267–1278. 10.1111/febs.13987 27973739

[B65] Ng A. N. Y. de Jong-Curtain T. A. Mawdsley D. J. White S. J. Shin J. Appel B. (2005). Formation of the Digestive System in Zebrafish: III. Intestinal Epithelium Morphogenesis. Dev. Biol. 286, 114–135. 10.1016/j.ydbio.2005.07.013 16125164

[B66] Nichols G. E. Shiraishi T. Allietta M. Tillack T. W. Young W. W. (1987). Polarity of the Forssman Glycolipid in MDCK Epithelial Cells. Biochimica Biophysica Acta (BBA) - Mol. Cell Res. 930, 154–166. 10.1016/0167-4889(87)90027-9 3040119

[B67] Odenwald M. A. Turner J. R. (2017). The Intestinal Epithelial Barrier: A Therapeutic Target? Nat. Rev. Gastroenterol. Hepatol. 14, 9–21. 10.1038/nrgastro.2016.169 27848962 PMC5554468

[B68] Pece S. Chiariello M. Murga C. Gutkind J. S. (1999). Activation of the Protein Kinase Akt/PKB by the Formation of E-Cadherin-Mediated Cell-Cell Junctions.Evidence for the Association of Phosphatidylinositol 3-Kinase With the E-Cadherin Adhesion Complex. J. Biol. Chem. 274, 19347–19351. 10.1074/jbc.274.27.19347 10383446

[B69] Pemberton J. G. Kim Y. J. Humpolickova J. Eisenreichova A. Sengupta N. Toth D. J. (2020). Defining the Subcellular Distribution and Metabolic Channeling of Phosphatidylinositol. J. Cell Biol. 219, 6130. 10.1083/jcb.201906130 PMC7054996 32211894

[B70] Pescio L. G. Favale N. O. Márquez M. G. Sterin-Speziale N. B. (2012). Glycosphingolipid Synthesis Is Essential for MDCK Cell Differentiation. Biochimica Biophysica Acta (BBA) - Mol. Cell Biol. Lipids 1821, 884–894. 10.1016/j.bbalip.2012.02.009 22387616

[B71] Polgar N. Fogelgren B. (2018). Regulation of Cell Polarity by Exocyst-Mediated Trafficking. Cold Spring Harb. Perspect. Biol. 10. 10.1101/cshperspect.a031401 PMC5587355 28264817

[B72] Rodriguez-Boulan E. Macara I. G. (2014). Organization and Execution of the Epithelial Polarity Programme. Nat. Rev. Mol. Cell Biol. 15, 225–242. 10.1038/nrm3775 24651541 PMC4211427

[B73] Roignot J. Peng X. Mostov K. (2013). Polarity in Mammalian Epithelial Morphogenesis. Cold Spring Harb. Perspect. Biol. 5, a013789. 10.1101/cshperspect.a013789 23378592 PMC3552506

[B74] Román-Fernández Á. (2018). The Phospholipid PI(3,4)P2 Is an Apical Identity Determinant. Nat. Commun. 9, 5041. 10.1038/s41467-018-07464-8 30487552 PMC6262019

[B75] Sampaio J. L. Gerl M. J. Klose C. Ejsing C. S. Beug H. Simons K. (2011). Membrane Lipidome of an Epithelial Cell Line. Proc. Natl. Acad. Sci. U.S.A. 108, 1903–1907. 10.1073/pnas.1019267108 21245337 PMC3033259

[B76] Sanchez T. Thangada S. Wu M.-T. Kontos C. D. Wu D. Wu H. (2005). PTEN as an Effector in the Signaling of Antimigratory G Protein-Coupled Receptor. Proc. Natl. Acad. Sci. U.S.A. 102, 4312–4317. 10.1073/pnas.0409784102 15764699 PMC555509

[B77] Sartorel E. Ünlü C. Jose M. Massoni-Laporte A. Meca J. Sibarita J.-B. (2018). Phosphatidylserine and GTPase Activation Control Cdc42 Nanoclustering to Counter Dissipative Diffusion. MBoC 29, 1299–1310. 10.1091/mbc.e18-01-0051 29668348 PMC5994902

[B78] Sasaki T. Takasuga S. Sasaki J. Kofuji S. Eguchi S. Yamazaki M. (2009). Mammalian Phosphoinositide Kinases and Phosphatases. Prog. Lipid Res. 48, 307–343. 10.1016/j.plipres.2009.06.001 19580826

[B79] Schlüter M. A. Margolis B. (2009). Apical Lumen Formation in Renal Epithelia. J. Am. Soc. Nephrol. 20, 1444–1452. 10.1681/ASN.2008090949 19497970

[B80] Schlüter M. A. Margolis B. (2012). Apicobasal Polarity in the Kidney. Exp. Cell Res. 318, 1033–1039. 10.1016/j.yexcr.2012.02.028 22421511 PMC3334475

[B81] Sedzinski J. Hannezo E. Tu F. Biro M. Wallingford J. B. (2016). Emergence of an Apical Epithelial Cell Surface *In Vivo* . Dev. Cell 36, 24–35. 10.1016/j.devcel.2015.12.013 26766441 PMC4735878

[B82] Shewan A. Eastburn D. J. Mostov K. (2011). Phosphoinositides in Cell Architecture. Cold Spring Harb. Perspect. Biol. 3, a004796. 10.1101/cshperspect.a004796 21576256 PMC3140688

[B83] Shi X. Gillespie P. G. Nuttall A. L. (2007). Apical Phosphatidylserine Externalization in Auditory Hair Cells. Mol. Membr. Biol. 24, 16–27. 10.1080/09687860600926883 17453410

[B84] Shlomovitz I. Speir M. Gerlic M. (2019). Flipping the Dogma - Phosphatidylserine in Non-apoptotic Cell Death. Cell Commun. Signal. 17, 139. 10.1186/s12964-019-0437-0 31665027 PMC6819419

[B85] Sigurbjörnsdóttir S. Mathew R. Leptin M. (2014). Molecular Mechanisms of De Novo Lumen Formation. Nat. Rev. Mol. Cell Biol. 15, 665–676. 10.1038/nrm3871 25186133

[B86] Skwarek L. C. Boulianne G. L. (2009). Great Expectations for PIP: Phosphoinositides as Regulators of Signaling during Development and Disease. Dev. Cell 16, 12–20. 10.1016/j.devcel.2008.12.006 19154715

[B87] Sohn M. Balla T. (2016). Lenz-Majewski Syndrome: How a Single Mutation Leads to Complex Changes in Lipid Metabolism. J. Rare Dis. Res. Treat. 2, 47–51. 10.29245/2572-9411/2017/1.1080 30854527 PMC6404757

[B88] Takahama S. Hirose T. Ohno S. (2008). aPKC Restricts the Basolateral Determinant PtdIns(3,4,5)P3 to the Basal Region. Biochem. Biophysical Res. Commun. 368, 249–255. 10.1016/j.bbrc.2008.01.083 18230334

[B89] Tan J. Oh K. Burgess J. Hipfner D. R. Brill J. A. (2014). PI4KIIIα Is Required for Cortical Integrity and Cell Polarity during Drosophila Oogenesis. J. Cell Sci. 127, 954–966. 10.1242/jcs.129031 24413170

[B90] Terawaki S. Maesaki R. Hakoshima T. (1993). Structural Basis for NHERF Recognition by ERM Proteins. Structure 14, 777–789. 10.1016/j.str.2006.01.015 16615918

[B91] Trotter P. J. Wu W.-I. Pedretti J. Yates R. Voelker D. R. (1998). A Genetic Screen for Aminophospholipid Transport Mutants Identifies the Phosphatidylinositol 4-kinase, STT4p, as an Essential Component in Phosphatidylserine Metabolism. J. Biol. Chem. 273, 13189–13196. 10.1074/jbc.273.21.13189 9582361

[B92] van Meer G. Voelker D. R. Feigenson G. W. (2008). Membrane Lipids: where They Are and How They Behave. Nat. Rev. Mol. Cell Biol. 9, 112–124. 10.1038/nrm2330 18216768 PMC2642958

[B93] van Zeijl L. Ponsioen B. Giepmans B. N. G. Ariaens A. Postma F. R. Várnai P. (2007). Regulation of Connexin43 Gap Junctional Communication by Phosphatidylinositol 4,5-bisphosphate. J. Cell Biol. 177, 881–891. 10.1083/jcb.200610144 17535964 PMC2064287

[B94] Wang Y. J. Wang J. Sun H. Q. Martinez M. Sun Y. X. Macia E. (2003). Phosphatidylinositol 4 Phosphate Regulates Targeting of Clathrin Adaptor AP-1 Complexes to the Golgi. Cell 114, 299–310. 10.1016/s0092-8674(03)00603-2 12914695

[B95] Wattelet-Boyer V. Brocard L. Jonsson K. Esnay N. Joubès J. Domergue F. (2016). Enrichment of Hydroxylated C24- and C26-Acyl-Chain Sphingolipids Mediates PIN2 Apical Sorting at Trans-golgi Network Subdomains. Nat. Commun. 7, 12788. 10.1038/ncomms12788 27681606 PMC5056404

[B96] Watton S. J. Downward J. (1999). Akt/PKB Localisation and 3′ Phosphoinositide Generation at Sites of Epithelial Cell-Matrix and Cell-Cell Interaction. Curr. Biol. 9, 433–436. 10.1016/s0960-9822(99)80192-4 10226029

[B97] Weernink P. A. O. Schulte P. Guo Y. Wetzel J. Amano M. Kaibuchi K. (2000). Stimulation of Phosphatidylinositol-4-Phosphate 5-Kinase by Rho-Kinase. J. Biol. Chem. 275, 10168–10174. 10.1074/jbc.275.14.10168 10744700

[B98] Willenborg C. Jing J. Wu C. Matern H. Schaack J. Burden J. (2011). Interaction between FIP5 and SNX18 Regulates Epithelial Lumen Formation. J. Cell Biol. 195, 71–86. 10.1083/jcb.201011112 21969467 PMC3187708

[B99] Xu R. Spencer V. A. Groesser D. L. Bissell M. J. (2010). Laminin Regulates PI3K Basal Localization and Activation to Sustain STAT5 Activation. Cell Cycle 9, 4315–4322. 10.4161/cc.9.21.13578 20980837 PMC3055185

[B100] Yan Y. Denef N. Tang C. Schüpbach T. (2011). Drosophila PI4KIIIalpha Is Required in Follicle Cells for Oocyte Polarization and Hippo Signaling. Dev. Camb. Engl. 138, 1697–1703. 10.1242/dev.059279 PMC3074446 21429988

[B101] Zegers M. M. P. O'Brien L. E. Yu W. Datta A. Mostov K. E. (2003). Epithelial Polarity and Tubulogenesis *In Vitro* . Trends Cell Biol. 13, 169–176. 10.1016/s0962-8924(03)00036-9 12667754

[B102] Zewe J. P. Miller A. M. Sangappa S. Wills R. C. Goulden B. D. Hammond G. R. V. (2020). Probing the Subcellular Distribution of Phosphatidylinositol Reveals a Surprising Lack at the Plasma Membrane. J. Cell Biol. 219, 6127. 10.1083/jcb.201906127 PMC7054989 32211893

[B103] Zhang H. Abraham N. Khan L. A. Hall D. H. Fleming J. T. Göbel V. (2011). Apicobasal Domain Identities of Expanding Tubular Membranes Depend on Glycosphingolipid Biosynthesis. Nat. Cell Biol. 13, 1189–1201. 10.1038/ncb2328 21926990 PMC3249144

[B104] Zhao H. Shiue H. Palkon S. Wang Y. Cullinan P. Burkhardt J. K. (2004). Ezrin Regulates NHE3 Translocation and Activation after Na + -glucose Cotransport. Proc. Natl. Acad. Sci. U.S.A. 101, 9485–9490. 10.1073/pnas.0308400101 15197272 PMC439003

[B105] Zhu L. Crothers J. Zhou R. Forte J. G. (2010). A Possible Mechanism for Ezrin to Establish Epithelial Cell Polarity. Am. J. Physiol. Cell Physiol. 299, C431–C443. 10.1152/ajpcell.00090.2010 20505040 PMC2928635

[B106] Zurzolo C. Simons K. (2016). Glycosylphosphatidylinositol-anchored Proteins: Membrane Organization and Transport. Biochimica Biophysica Acta (BBA) - Biomembr. 1858, 632–639. 10.1016/j.bbamem.2015.12.018 26706096

